# Non-dilation endoscopic ultrasound-guided hepaticoduodenostomy and hepaticogastrostomy using a 7-Fr delivery system

**DOI:** 10.1055/a-2796-7662

**Published:** 2026-02-27

**Authors:** Hidenobu Hara, Yoko Henta, Risa Katsumata, Hiroaki Matsumoto, Shiori Ito, Kouhei Yoshino, Shinya Sakita

**Affiliations:** 1 53327 Department of Gastroenterology, Yokohama City Minato Red Cross Hospital, Yokohama, Japan

**Fig. 1:**
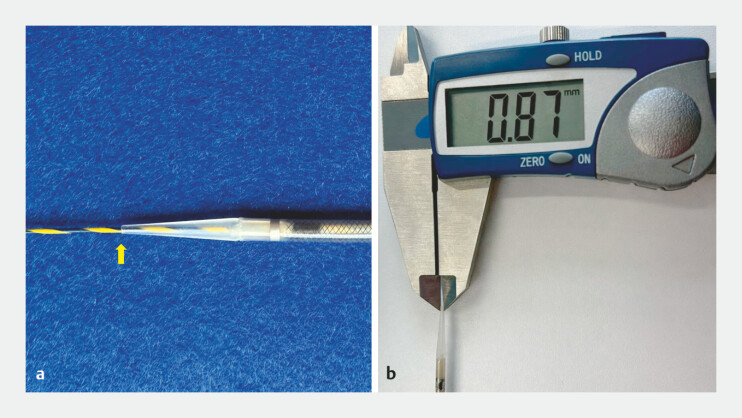
7-Fr delivery partially covered slim-delivery self-expandable metal stents used in the procedure. **a** A 0.025-inch guidewire and the 7-Fr delivery catheter (arrow). **b** The ultra-tapered distal tip measures 0.87 mm (approximately 2.6 Fr).

Transpapillary drainage may be inadequate for malignant hilar biliary obstruction with extensively separated ducts, and endoscopic ultrasound guided biliary drainage (EUS-BD) has been reported as salvage therapy 1 2 3 . 7-Fr slim-delivery self-expandable metal stents (SEMSs) have enabled non-dilation EUS-BD ( 4 5 ; Fig. 1 ). We report jaundice relief using non-dilation EUS-guided hepaticoduodenostomy and hepaticogastrostomy (EUS-HDGS) with a 7-Fr delivery partially covered SEMS system for hepatocellular carcinoma–related hilar obstruction.

**Fig. 2:**
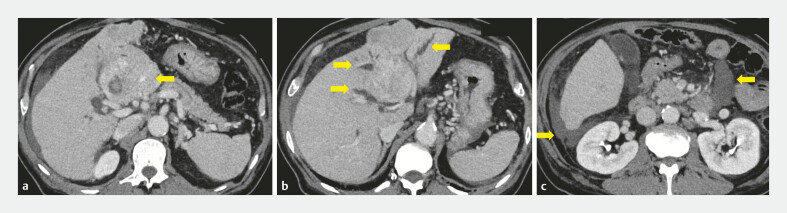
Pre-procedural contrast-enhanced computed tomography. **a** Intratumoral hemorrhage in hepatocellular carcinoma (arrow). **b** Malignant hilar biliary obstruction with separate intrahepatic ducts (arrow). **c** Moderate ascites due to cirrhosis (arrow).

**Fig. 3:**
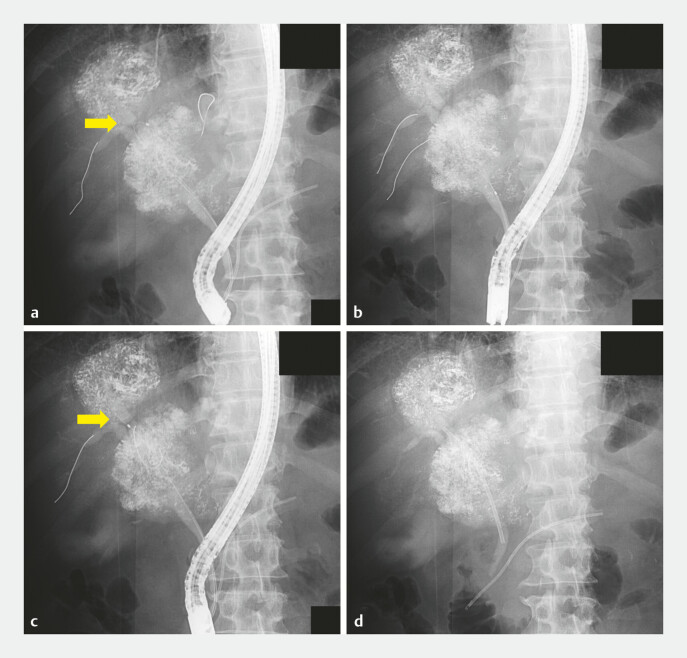
Fluoroscopic images during endoscopic retrograde cholangiopancreatography. **a** Guidewires were advanced into B3 and B5; catheter advancement toward B3 was impeded by sharp angulation (arrow). **b** The guidewires were advanced into B5 and B6. **c** Catheter advancement into B6 was not possible (arrow). **d** An internal plastic stent was placed in the anterior sectoral duct, and the procedure was completed.

A 68-year-old man with hepatitis C-related cirrhosis (Child-Pugh score, 11) and moderate ascites exhibited malignant hilar obstruction with separated ducts due to hepatocellular carcinoma. Because the tumor ruptured, emergency transarterial embolization was prioritized ( Fig. 2 ). Endoscopic retrograde cholangiopancreatography was attempted, and although guidewire access was achieved, device advancement, including a catheter, was impossible due to severe stenosis and marked ductal deviation. Therefore, an internal plastic stent (7 Fr, 9 cm) was placed only in the anterior sectoral duct ( Fig. 3 ). However, additional biliary drainage was required due to persistent jaundice, and EUS-HDGS was planned ( Video 1 ).

EUS-guided hepaticoduodenostomy and hepaticogastrostomy for hilar obstruction with separated ducts. EUS, endoscopic ultrasound.Video 1

**Fig. 4:**
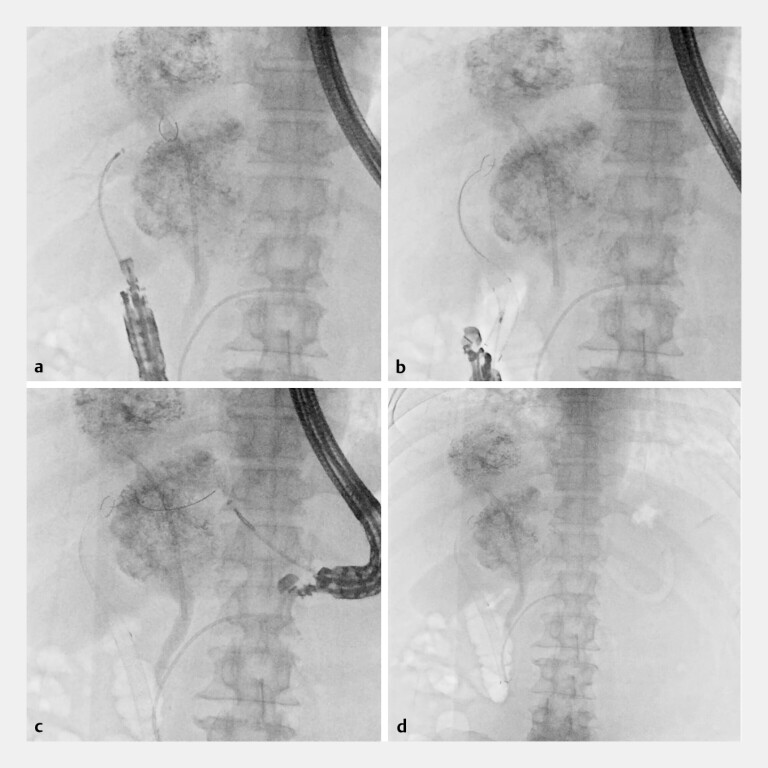
Fluoroscopic images during endoscopic ultrasound-guided hepaticoduodenostomy and hepaticogastrostomy. **a** A 7-Fr delivery catheter was advanced from the duodenal bulb into B6 without tract dilation. **b** A partially covered SEMS (8 mm × 12 cm) was deployed from B6 to the duodenal bulb. **c** A 7-Fr delivery catheter was advanced from the stomach into B3 without dilation. **d** Partially covered SEMSs (8 mm × 12 cm) were deployed from B3 to the stomach. SEMS, self-expandable metal stent.

From the duodenal bulb, B6 was punctured using a 19-gauge FNA needle, and a 0.025-inch guidewire was advanced into the intrahepatic duct. After bile aspiration and cholangiography, a partially covered SEMS (8 mm × 12 cm) with a 7-Fr delivery system was deployed without tract dilation ( Fig. 4 **a, b** ). B3 was similarly accessed from the stomach. A second partially covered SEMS (8 mm × 12 cm) was placed without tract dilation ( Fig. 4 **c, d** ). Postprocedural computed tomography confirmed appropriate stent positions. No adverse events occurred, and total bilirubin levels decreased by >50% by day 6.

In patients with advanced cirrhosis and ascites, minimizing tract manipulation is desirable due to concerns regarding bleeding and bile leakage. A 7-Fr delivery system may facilitate non-dilation and multiroute EUS-BD for malignant hilar obstructions with separated ducts.

Endoscopy_UCTN_Code_TTT_1AS_2A
